# Network Influence vs. Credibility in YouTube Sleep‐Health Communication

**DOI:** 10.1002/puh2.70251

**Published:** 2026-04-27

**Authors:** Atousa Ghahramani, Maria Prokofieva, Maximilian Pangratius de Courten

**Affiliations:** ^1^ Institute for Sustainable Industries & Liveable Cities Business School Victoria University Melbourne Australia; ^2^ Institute for Health and Sport and Australian Health Policy Collaboration Victoria University Melbourne Australia

**Keywords:** algorithmic visibility, credibility, digital health, health communication, networked influence, social media platforms, YouTube

## Abstract

Insomnia is a prevalent sleep disorder that remains widely underdiagnosed and undertreated, prompting many individuals to seek health information beyond formal healthcare systems. Social media platforms, such as YouTube, have become influential spaces for the circulation of sleep‐health information; however, concerns persist regarding whether credible, expert‐led content achieves sufficient visibility to support population‐level health outcomes. This study analyses 98 English‐language YouTube videos to examine how sleep‐health information circulates within an algorithmically mediated environment, focusing on the relationship between source credibility, network visibility, audience engagement and diffusion potential. Drawing on diffusion of innovations and social cognitive theory, the study conceptualises YouTube as a sociotechnical system in which opinion leadership, social reinforcement and algorithmic amplification jointly shape influence. The findings revealed a systematic credibility–influence gap, whereby visibility and diffusion are driven more by network position, engagement dynamics and algorithmic amplification than by source credibility. The results highlight a key public health challenge in which credible sleep‐health information struggles to achieve reach within platform‐mediated systems. By identifying the network and engagement mechanisms that shape diffusion, this study provided evidence to inform more effective digital health communication strategies aimed at increasing the visibility of trustworthy sleep‐health information and supporting healthier lifestyles.

## Graphical abstract

This study shows that the visibility and diffusion of sleep‐health information on YouTube are driven primarily by network position, audience engagement and algorithmic amplification rather than source credibility. A credibility–influence gap emerges, where highly engaging content achieves greater reach regardless of expertise, highlighting a key challenge for public health communication in platform‐mediated environments.

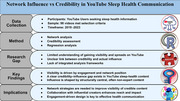

## 1 Introduction

Insomnia is identified as one of the most prevalent sleep disorders that affected more than 16% of the global population [1]. Despite its high prevalence, insomnia remains underdiagnosed and undertreated, with many individuals facing barriers to accessing formal and trustworthy sleep‐health services, in addition to the gaps in health literacy that limit their ability to understand and critically evaluate sleep‐health‐related information [2]. Its implications for individual healthcare and well‐being, along with the increased risk of chronic conditions such as cardiovascular disease and mental health disorders, emphasise the urgent need for public health researchers and practitioners to promote and strengthen evidence‐based sleep‐health behaviours within communities [3, 4]. The impact of insomnia extends beyond individual health outcomes; it represents a substantial population‐level cost, associated with an increased risk of chronic conditions and measurable productivity losses. Economic analysis revealed that insufficient sleep leads to absenteeism, presenteeism and decreased workplace performance (Hillmand et al. 2018) [5, 6]. These data position insomnia as a systemic public health issue requiring scalable, population‐level communication and intervention strategies [7].

These challenges contribute to a persistent treatment gap, prompting individuals to seek alternative sources of information outside traditional healthcare systems through using digital environments for guidance on self‐management of insomnia [8].

In this context, digital media platforms have increasingly influenced how individuals access and interpret health‐related information [9]. Among these platforms, YouTube plays a significant role due to its global accessibility and participatory nature, enabling users not only to consume content but also to actively engage in discussions around sleep‐health‐related information [10]. Through social interaction and the sharing of personal experiences, YouTube can shape health‐related behaviours and perceptions by enabling users to observe and evaluate others’ practices while also engaging with the contents through likes, comments and discussions [2, 11].

Beyond its social affordances, YouTube can be understood as a sociotechnical system in which social interaction, network structures and algorithmic processes jointly shape the circulation of information [12]. Algorithmic recommendation systems of YouTube further shape information exposure based on the patterns of user behaviour and engagement and the dynamics of the network. Consequently, YouTube functions as an active, algorithmically mediated environment that influences how health‐related information is disseminated within the platform [13, 14, 15, 16].

The emerging research highlighted both the opportunities and risks associated with YouTube as a source of health‐related information. Although the platform has the potential to support public awareness [17] and facilitate the large‐scale dissemination of evidence‐based health‐related content [18], concerns persist regarding the quality, accuracy and credibility of such content, as misleading or commercially driven information can also achieve high visibility [19, 20, 21, 22, 23]. Although expert‐led YouTube videos offer clinically grounded guidance [17], the widespread presence of misinformation may undermine public health efforts and encourage harmful behaviours [10, 24].

Existing studies have focused on evaluating the quality and reliability of health information on YouTube through assessing the prevalence of misinformation using tools such as DISCERN and the global quality scale (GQS) [25, 26]. Although these approaches provide insights to the accuracy of the content, there are limitations in assessing how information achieves visibility and influence within the platform. There is a critical gap in understanding how credible health information circulates within a particular digital network alongside more engaging content and less credible information. Moreover, limited research has examined how credible sleep‐health information circulates and achieves influence on social media platforms [2].

### 1.1 Problem Statement

A key unresolved issue is whether credible, evidence‐based sleep‐health information can achieve sufficient visibility and influence within algorithmically mediated platforms, such as YouTube. In such environments, engagement‐driven and network‐based dynamics may prioritise content irrespective of its credibility. This raises an important concern in the context of public health, as the decoupling of visibility and influence from credibility may structurally disadvantage the dissemination of reliable health information.

To address this problem, it is necessary to move beyond the evaluation of shared content and examine the structural and behavioural mechanisms that shape the diffusion of health‐related information within digital environments, such as YouTube [27].

Drawing on diffusion of innovations (DOI) and social cognitive theory (SCT), this study supported a theoretically grounded framework for analysing how influence emerges in platform‐mediated contexts. DOI conceptualises influence as shaped by opinion leaders and change agents, whose visibility and authority affect the adoption of information, particularly when they occupy central positions within social networks [28]. Complementing this perspective, SCT highlighted the role of observational learning, social reinforcement and perceived norms in shaping how audiences interpret, engage with and respond to content [29]. In algorithmically mediated environments such as YouTube, these processes are further intensified, as recommendation systems prioritise content based on engagement and network position rather than source credibility alone [16, 30, 31].

Although existing research has documented the presence of misinformation and variability in content quality on YouTube, limited studies examined the relationship between engagement, source credibility and networked influence, particularly in the context of sleep‐health communication. Understanding this relationship is critical for public health practitioners, as it determines whether credible information can effectively reach and influence audiences at the population level.

This study contributed to knowledge through addressing this gap by examining how sleep‐health information circulates within YouTube's sociotechnical environment, focusing on the relationship between source credibility, network visibility, audience engagement and diffusion potential. By integrating DOI and SCT with social network analysis (SNA), the study conceptualised influence as emerging from structural positioning, social reinforcement and algorithmic processes. Within this framework, opinion leadership was measured using network centrality, whereas change agency was assessed through a source‐based credibility classification that distinguishes between expert and non‐expert creators. Engagement indicators, including likes, comments and replies, were used to capture processes of social reinforcement and audience interaction [29, 32, 33].

Two complementary network models were employed: an actor–network model to identify structurally central videos functioning as opinion leaders, and an activity‐network model to examine co‐engagement patterns that reflected observational learning and social reinforcement. Together, these approaches facilitated a systematic analysis of network structure, audience behaviour and algorithmic dynamics to shape the visibility and diffusion of sleep‐health information on YouTube.

Table 1 mapped key constructs from DOI and SCT to the empirical measures used in this analysis.

**TABLE 1:** Mapping diffusion of innovations (DOI) and social cognitive theory (SCT) constructs to study measures.

Construct	Theory	Operationalisation
**Opinion leadership**	DOI	Network centrality of videos (eigenvector, degree, betweenness)
**Change agency**	DOI	Source‐credibility classification based on creator type and professional/institutional affiliation
**Social structure/Diffusion pathways**	DOI	Actor–network linking videos, users and algorithmic recommendations
**Observational learning**	SCT	Co‐engagement patterns across videos (activity‐network)
**Social reinforcement**	SCT	Engagement signals (likes, comments, replies) as indicators of perceived social approval
**Diffusion potential**	DOI + SCT	Combined structural visibility and reinforcement patterns (centrality + clustering)

## 2 Methods

### 2.1 Study Design

This study employed a network‐analytic design to examine how sleep‐health information circulates within the YouTube ecosystem. The analysis focused on three empirically observable indicators of social influence: visibility, engagement and diffusion potential. Visibility captured the structural prominence of videos, including their centrality within interaction networks and exposure through algorithmic recommendation pathways. Engagement reflected observable audience interactions, likes, comments and replies, which function as behavioural cues of social reinforcement. Diffusion potential refers to a video's structural capacity to facilitate information flow across the network, operationalised through centrality measures within the actor–network.

Although persuasion, behavioural adoption and message effectiveness were theoretically relevant, they were not directly observable in platform‐level data and, therefore, were not examined. The analysis was intentionally limited to visibility, engagement, and diffusion potential as empirically grounded indicators of influence in an algorithmically mediated environment.

### 2.2 Data Collection

This study compiled a dataset of publicly available YouTube videos. Data were collected via the YouTube Data API using an academic developer account for videos published between January 2019 and December 2023 [16]. The search used validated keywords, ‘sleep’, ‘sleep behaviour’, ‘insomnia’, ‘sleep disorder’, ‘sleep habits’ and ‘sleep hygiene’, refined through Google Trends to ensure alignment with contemporary public search patterns.

From an initial set of 257 videos, a systematic screening process was applied to ensure analytical suitability. Videos were included if they were English‐language, focused on sleep health, and had active comment sections, whereas non‐English content, music or white‐noise videos and videos with disabled comments were excluded [34]. This process resulted in a final sample of 98 videos.

For each video, metadata and user‐interaction data (comments and replies) were collected to support network analysis, with creator subscriber counts included where available to contextualise network influence. Channels with hidden subscriber counts were retained. As of 30 December 2024, the dataset comprised 62,949,118 views, 41,574 comments and 22,288 replies and was analysed in RStudio using established network‐analytic workflows.

### 2.3 Credibility Scoring Framework for YouTube Video Sources

To assess the credibility of YouTube video sources, a source‐based scoring framework adapted from established criteria was applied [34]. Creators were categorised on a five‐point scale based on stated credentials and institutional affiliation, ranging from official medical or government institutions (score = 5) to non‐expert bloggers (score = 1). This research‐based framework provided an independent assessment of source credibility beyond platform‐driven signals, such as authoritative labels [35]. All sources were manually coded by two independent reviewers, with discrepancies resolved through discussion and, where necessary, third‐coder validation to ensure reliability [34].

### 2.4 Social Network Analysis

SNA was employed to examine how interactions among viewers, creators and content structure the circulation of sleep‐health information on YouTube. Networks were constructed by linking users and videos through interactional ties such as comments and replies, allowing analysis of communication concentration and audience organisation. Centrality and clustering measures were used to identify opinion‐leadership hubs and audience communities, linking network structure to diffusion processes and social learning within an algorithmically mediated environment.

## 3 Results

### 3.1 Analysis of Influential Actors Within the Network

Actor–network analysis was conducted to examine how interactions among users, creators, videos and algorithmic recommendation pathways shape the circulation of sleep‐health information on YouTube. Networks linked videos and users through comment‐based interactions, incorporating likes, replies and approximated recommendation ties to capture both human and algorithmic influences [36, 37]. Influential actors were identified using centrality measures, with eigenvector centrality used as the primary indicator of structural prominence [38].

**FIGURE 1:**
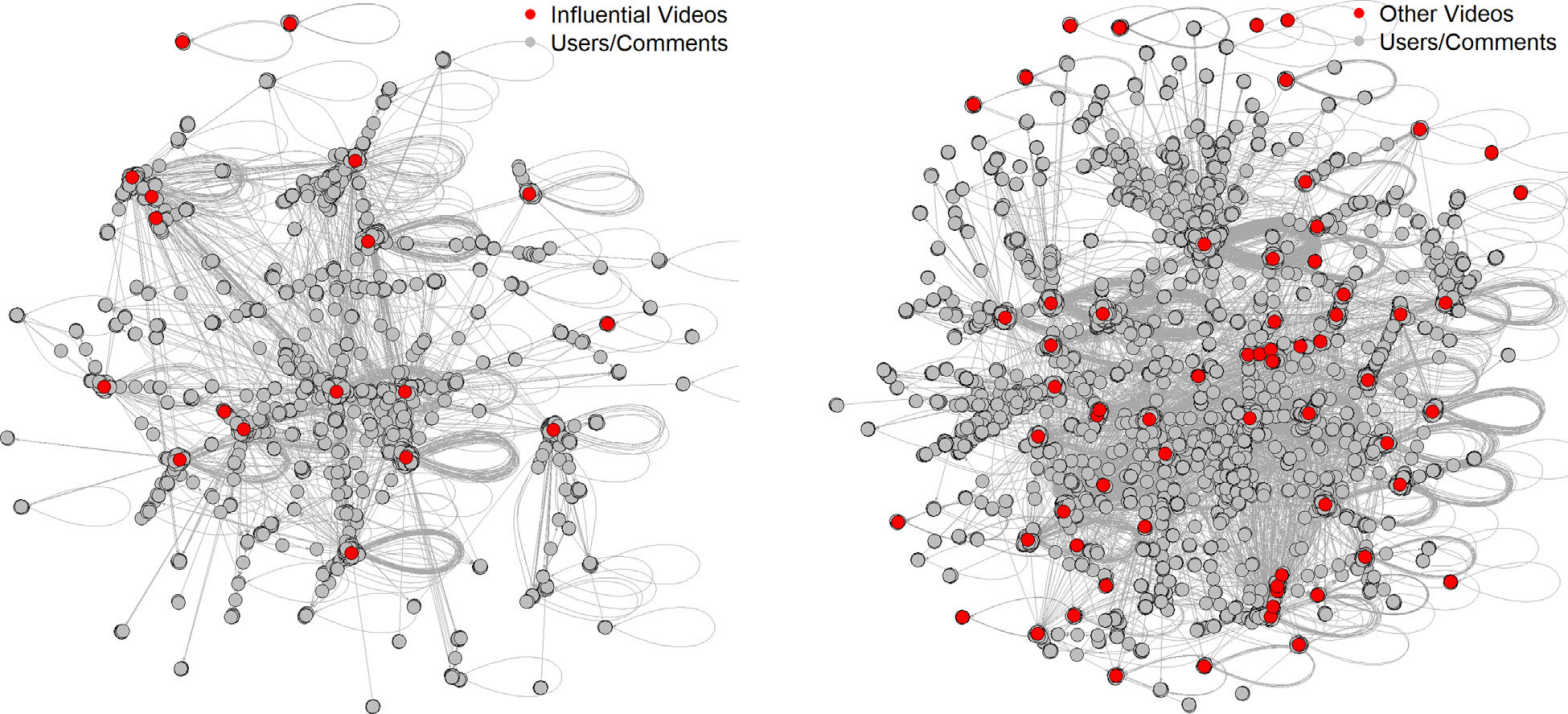
Actor–network analysis of 18 influential videos and 80 other videos.

As illustrated in Figure 1, 18 videos exceeded the centrality threshold and formed a highly centralised cluster (clustering coefficient = 0.66), functioning as opinion leaders consistent with DOI theory [39]. A centrality threshold is a predefined cut‐off applied to centrality scores to identify nodes with high network influence, with only those exceeding the threshold included in the highly central cluster [40]. Engagement ties reflected SCT mechanisms of observational learning and social reinforcement, whereas several influential videos originated from creators with relatively small subscriber bases, indicating that structural positioning and algorithmic visibility, rather than pre‐existing popularity, shaped influence.

### 3.2 Audience Interactions

Audience interaction patterns were denser around influential videos, with higher interaction intensity (27.06 edges per node) compared to other videos (24.66), indicating stronger communication channels (Table 2). From a DOI perspective, these dense networks facilitate faster diffusion through reinforced relational pathways, whereas from an SCT perspective, high engagement functions as social reinforcement that promotes observational learning. Influential videos fostered more cohesive and interconnected audience communities, whereas non‐influential videos exhibited broader but more fragmented interaction structures, diffusing attention across loosely connected groups.

**TABLE 2:** Descriptive comparison of actor–networks.

Metrics	Influential videos (*n* = 18)	Other videos (*n* = 80)	Interpretation
Edges per node	27.06	24.66	Influential videos show slightly denser interaction patterns
Clustering coefficient	0.66	0.66	Both groups display similar levels of local clustering
Engagement structure	Cohesive, interconnected clusters	More fragmented clusters	Influential videos attract more structurally cohesive engagement
Diffusion potential	Higher, due to stronger network embeddedness	Moderate, with broader but less coordinated spread	Structural positioning gives influential videos greater diffusion capacity

### 3.3 Distribution of Source Credibility Across Videos

A Chi‐square test was conducted separately for influential and other videos to assess whether the distribution of source credibility categories differed from a uniform distribution (Table 3). For the other videos (*n* = 80), the test was statistically significant, *χ* ^2^(4, *N* = 80) = 124.88, *p* < 0.001, indicating a highly uneven distribution of credibility scores. As shown in Table 3, this group was heavily concentrated in mid‐level credibility categories, particularly health experts (credibility score = 3), whereas the highest‐credibility sources, such as official medical or government institutions, were absent.

**TABLE 3:** Distribution of YouTube video sources promoting sleep‐health behaviours.

		Other videos (*n* = 80)		Influential videos (*n* = 18)	
Source type	Credibility score	No. of videos	Source type (%)	No. of videos	Source type (%)
Sleep‐health institutes	5	*n* = 0	0.0	*n* = 2	100
Sleep‐health professional	4	*n* = 3	50	*n* = 3	50
Health experts	3	*n* = 55	87.3	*n* = 8	12.7
News sources	2	*n* = 10	76.9	*n* = 3	23.1
Blogger	1	*n* = 12	85.7	*n* = 2	14.3

*Note:* Credibility score of source: 5 = official government or medical institution (e.g., Sleep Foundation and Psych Hub); 4 = recognised sleep‐health professionals (sleep coaches, neuroscientists and sleep researchers); 3 = health experts (psychologists, psychiatrists, medical doctors and family therapists); 2 = reputable news organisations (e.g., The Guardian, ABC News and WPLG Local); 1 = bloggers or creators without verifiable expertise (higher risk of misinformation). **Other videos**: all videos (98)—influential videos (18).

In contrast, for influential videos (*n* = 18), the Chi‐square test was not statistically significant, *χ* ^2^(4, *N* = 18) = 7.00, *p* = 0.136, suggesting no strong deviation from a uniform distribution. Influential videos were more evenly distributed across credibility categories, with representation from high‐, mid‐, and lower credibility sources, and no single category dominating the content.

### 3.4 Linking Credibility Assessment to DOI Theory

Analysis of source credibility and network position showed that influential videos included both expert and non‐expert creators. Non‐expert creators were frequently structurally central within the network, whereas expert and institutional sources were less consistently positioned for visibility. Although official sleep‐health institutions appeared among influential videos, higher credibility was not associated with higher engagement levels. As shown in Table 4, influential videos were distributed across credibility categories, indicating that network positioning rather than source credibility alone was associated with diffusion potential.

**TABLE 4:** Mapping credibility assessment to diffusion of innovations (DOI) constructs.

DOI role	How DOI defines it	How it appears on YouTube	Operational measure
Opinion leaders	Non‐experts embedded in social networks; highly trusted; consume more media; shape peer exposure	Highly connected creators or videos that achieve structural visibility regardless of expertise	Network centrality (eigenvector, degree and betweenness)
Change agents	Experts or institutions who promote evidence‐based innovations and reduce uncertainty	Health professionals, medical institutions, sleep researchers	Source‐based credibility classification

### 3.5 Methodological Benchmarking of Source‐Based Credibility Against DISCERN and GQS

To assess robustness and external validity, the source‐based credibility framework was benchmarked against the DISCERN index and the GQS, two established instruments for evaluating health information quality [34, 41, 42]. Although DISCERN and GQS assess informational quality through expert review at the video level, the framework used in this study captured structural source credibility based on creator affiliation and professional accountability. Together, these approaches represent complementary dimensions of credibility, distinguishing between informational accuracy and structural trust. The source‐based framework was operationalised as an ordinal measure using non‐parametric tests and offers a scalable alternative suitable for large‐scale network analysis.

### 3.6 Relationship Between Source Credibility and Engagement Level

**FIGURE 2:**
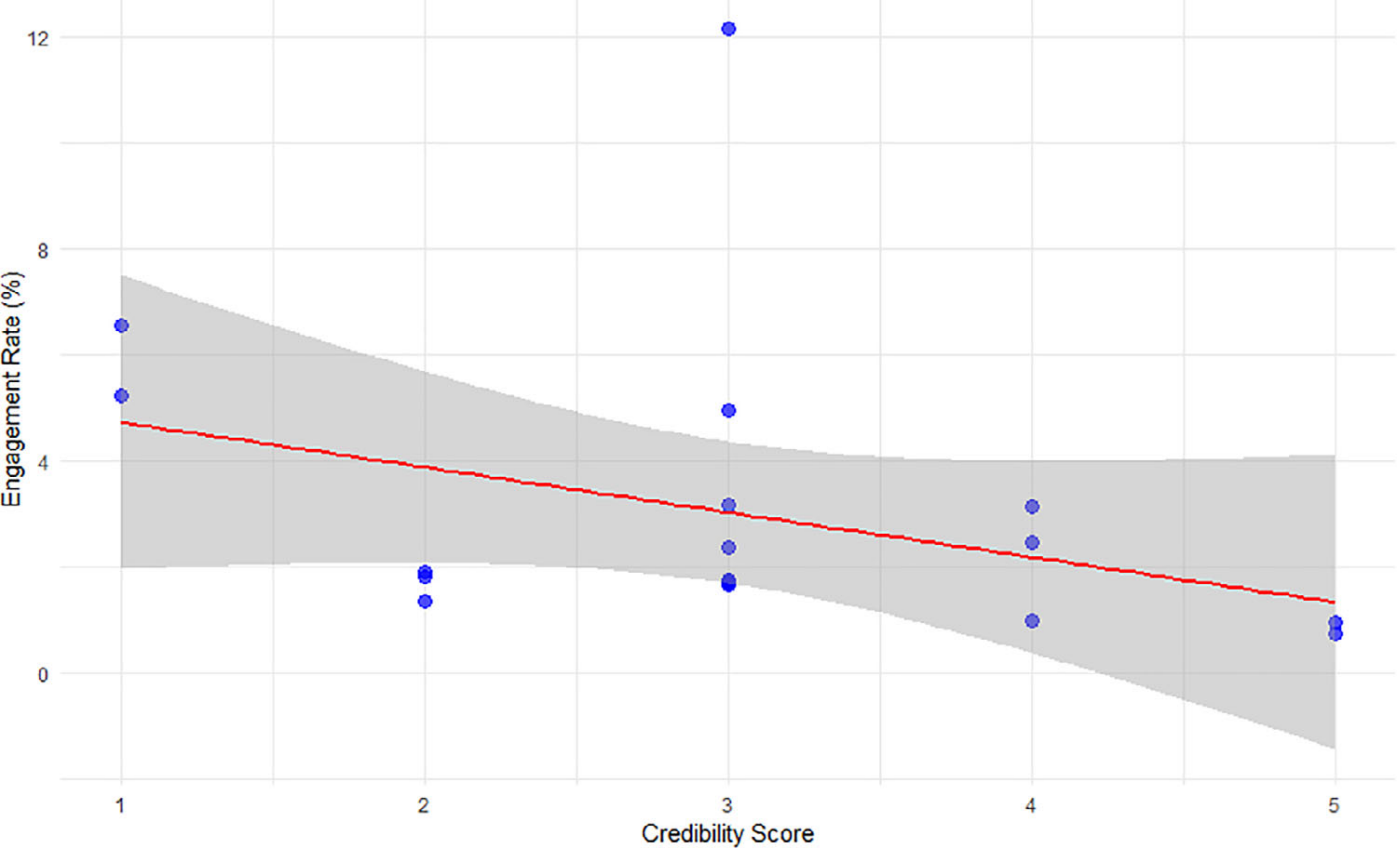
Engagement rate versus credibility in influential videos.

**FIGURE 3:**
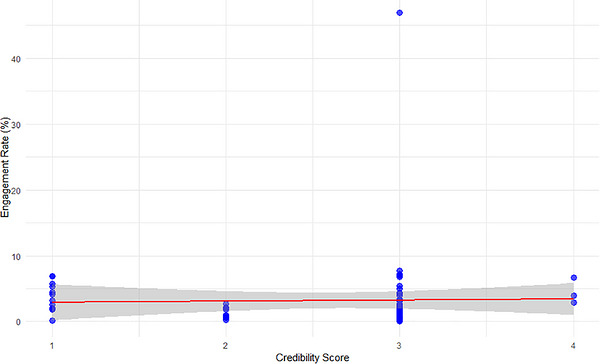
Engagement rate versus credibility in the other videos.

Among influential videos, source credibility showed a small negative but non‐significant association with engagement (Spearman's *ρ* = –0.21, 95% CI: −0.47 to 0.09, *p* = 0.16), whereas among other videos, the association was effectively null (*ρ* = 0.02, 95% CI: −0.12 to 0.15, *p* = 0.83) (Figures 2 and 3). These results indicated that user engagement on YouTube is largely independent of source‐level credibility, reinforcing a credibility–influence gap in which structurally visible or algorithmically amplified content was not necessarily produced by more credible sources. This pattern aligns with prior research using DISCERN and GQS, which similarly demonstrates that engagement is a poor proxy for informational quality.

Credibility assessment further showed that official health institutions (credibility score = 5) appeared only among influential videos, consistent with their role as DOI change agents; however, higher credibility did not reliably translate into greater engagement. From an SCT perspective, highly engaging but less credible content may attract stronger attention and social reinforcement when expertise is less visible or relatable. As shown in Table 3, influential videos are more likely to originate from higher‐credibility sources, although such sources do not dominate the overall dataset.

The presence of an outlier with exceptionally high engagement suggests that factors beyond source credibility, such as early interaction dynamics, algorithmic surfacing, or pre‐existing audience reach, may have disproportionately shaped visibility. This interpretation is consistent with evidence that YouTube's recommendation system amplifies content that attracts rapid initial engagement regardless of informational quality [43]. Engagement was operationalised as a normalised rate based on likes, comments and replies relative to total views, allowing comparison across videos with differing levels of exposure.

### 3.7 Analysis of User Interaction Patterns Within the Network

An activity network analysis was conducted to examine user interaction patterns in YouTube sleep‐health promotion, providing insights into content dissemination, engagement and interaction structures [44].

The network, constructed in R, used nodes for activities (video uploads, comments and replies) and edges for relationships (direct engagement or responses). Key metrics, degree centrality, betweenness centrality, clustering coefficient and network density, were calculated to evaluate connectivity, bridging roles, localised engagement and overall interactivity [45].

### 3.8 Activity Networks (Social Reinforcement and Community Structure)

Activity‐network analysis showed that influential videos generated stronger audience connectivity, with higher degree centrality (0.067 vs. 0.054) and greater clustering (0.684 vs. 0.631) than other videos. These tightly connected interaction structures enhance diffusion potential in DOI terms and reinforce SCT processes of social learning through repeated signals of audience approval. Influential videos formed denser and more cohesive discussion clusters, whereas other videos attracted broader but more fragmented engagement despite similar betweenness centrality.

**FIGURE 4:**
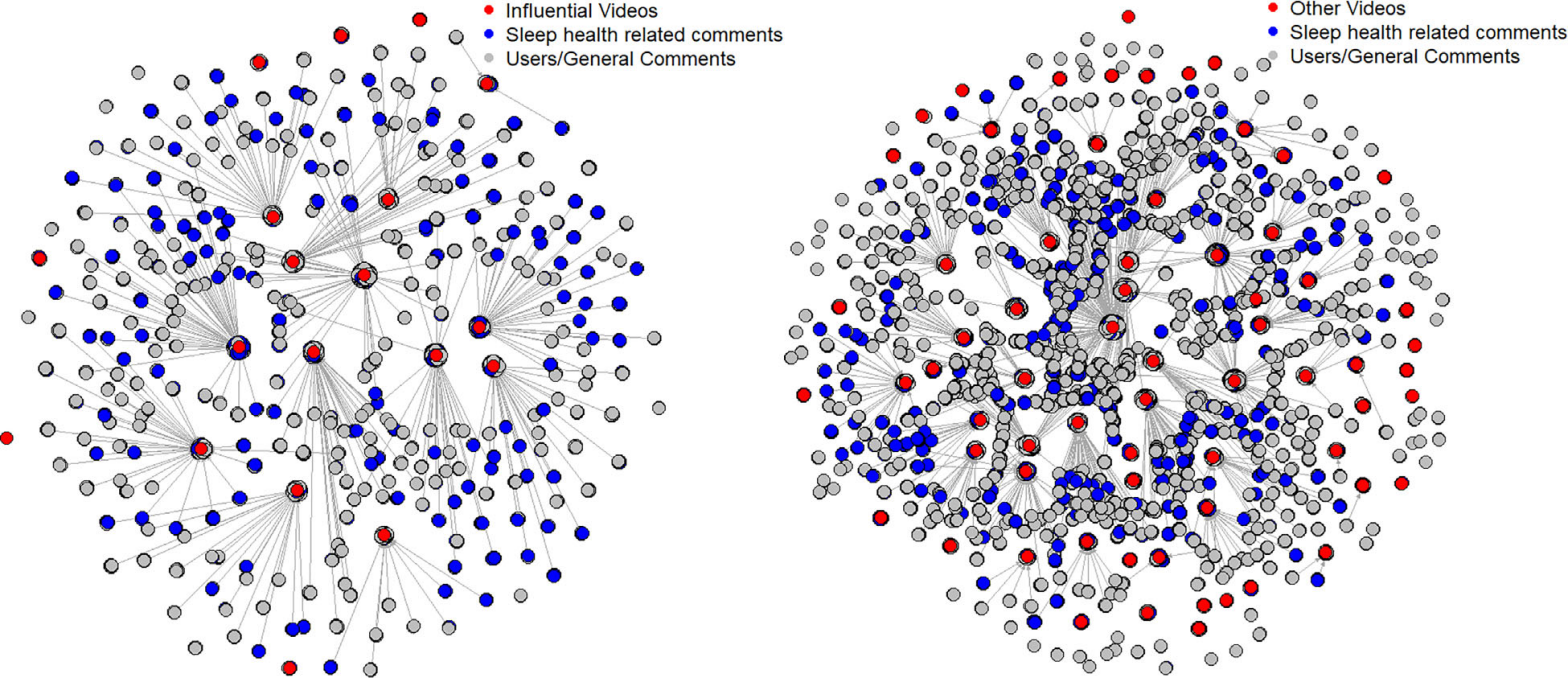
Activity network analysis of 18 influential videos and 80 other videos.

The activity‐network model audiences engage through co‐engagement ties between videos based on overlapping user interactions, distinguishing it from the actor–network, which captures broader structural positioning. This distinction highlights the complementary roles of structural influence and social reinforcement in shaping the diffusion of sleep‐health information. Figure 4 visualises these dynamics, with red nodes representing videos, blue nodes representing comments, grey nodes representing users and edges indicating interaction pathways.

Table 5 compared activity networks, showing that influential videos generate stronger user engagement and more cohesive interaction patterns, whereas other videos produce dispersed, fragmented discussions with weaker structural connectivity.

**TABLE 5:** Comparison of key metrics and aspects in activity networks.

Metrics and aspects	Influential videos (*n* = 18)	Other videos (*n* = 80)	Key insights
Edges per node	20.83	17.05	Influential videos exhibit higher interaction density, indicating more active co‐engagement among users
Degree centrality	0.067	0.054	Higher degree centrality suggests that influential videos attract a greater number of direct interactions
Betweenness centrality	0.0056	0.0057	Similar values across groups indicate comparable levels of intermediary positioning within the network
Clustering coefficient	0.684	0.631	Influential videos show stronger local clustering, forming more cohesive user‐interaction patterns
Connectivity	Denser interaction patterns	More dispersed interactions	Influential videos draw audiences into more interconnected structures
Community structure	More cohesive discussion clusters	Less cohesive, more diffuse clusters	Influential videos are embedded within tighter audience communities

## 4 Discussion

This study examined how sleep‐health information circulates on YouTube by analysing visibility, engagement and diffusion potential within an algorithmically mediated environment. The findings show a clear decoupling of credibility from influence: videos that achieved high visibility and diffusion potential were shaped primarily by network embeddedness, audience interaction and algorithmic amplification rather than by source credibility. Structurally central videos functioned as opinion leaders in the DOI sense, even when produced by non‐experts, whereas institutional and professional channels, despite high credibility, often failed to achieve comparable reach.

These findings extended DOI and SCT by demonstrating how their core mechanisms operate under platform conditions. In algorithmically governed environments, opinion leadership is driven less by expert authority and more by structural position within attention networks [31]. SCT helped explain this divergence: engagement signals such as likes, comments and replies acted as social reinforcement cues that elevated content visibility regardless of informational quality [46]. Although such metrics did not capture persuasion or behavioural change, they indicated audience responsiveness, which is central to diffusion potential in digital media systems [47].

The prominence of highly central videos from relatively unknown or newly established channels further suggested that influence on YouTube cannot be attributed solely to pre‐existing audience size [48, 49]. Instead, early engagement and subsequent algorithmic amplification appeared to play a decisive role in shaping visibility, consistent with preferential‐attachment dynamics that reward rapid initial interaction [50]. Together, these dynamics positioned YouTube as a sociotechnical ecosystem in which engagement‐driven and algorithmic logics privilege network prominence over epistemic authority, enabling highly engaging but less reliable content to circulate more widely than expert‐produced material [51].

Taken together, the results revealed a systemic credibility–influence gap in which visibility and opinion leadership were driven primarily by network positioning and algorithmic amplification rather than by the authority of credible change agents. This challenges the assumption within DOI that expertise and influence naturally align and illustrates how SCT‐related social reinforcement mechanisms can prioritise engagement and visibility over epistemic quality. By combining measures of network centrality, co‐engagement and engagement intensity, the study revealed that structurally prominent videos function as diffusion hubs regardless of credibility, indicating that influence in YouTube's sociotechnical environment emerges from network embeddedness and audience behaviour rather than creator credibility alone [52].

By theorising this credibility–influence gap, the study addressed a limitation in prior research on health information on YouTube, which has largely focused on documenting misinformation prevalence without explaining why credible content often struggles to gain reach [10]. Methodologically, the integrated use of actor–network analysis, activity‐network analysis, and source‐based credibility assessment provided a robust and scalable framework for examining how structural positioning, audience behaviour and platform infrastructures jointly shape the circulation of health information [34].

### 4.1 Implications for Public Health Communication

The findings have important implications for public health communication policy in platform‐mediated environments. Reliance on institutional authority alone is unlikely to ensure reach without strategies that account for network structure and early engagement dynamics. Public health agencies may therefore benefit from network‐informed dissemination approaches, including collaboration with structurally influential creators and the strategic design of messages to stimulate early interaction and algorithmic visibility. Integrating network diagnostics into digital health campaigns can support more targeted, efficient and policy‐responsive dissemination of credible health information, aligning public health objectives with platform‐specific dynamics.

### 4.2 Limitations of the Study and Recommendations for Future Research

This study has several limitations that should be acknowledged. First, the analysis relied on publicly available YouTube data, excluding private user information such as demographics, detailed viewing behaviour and long‐term engagement trajectories, which limits individual‐level inference [53]. The English‐language, keyword‐based sampling strategy may also reduce generalisability to non‐English‐speaking or niche communities.

Second, although the network and credibility analyses illuminate diffusion dynamics, the study did not incorporate experimental designs or user surveys to assess audience perceptions, trust or behavioural outcomes [54]. Credibility was operationalised at the source level, but identifying subtle misinformation or commercial bias often requires expert content‐level evaluation [55].

Third, the proprietary and evolving nature of YouTube's recommendation algorithms constrained the ability to model their effects on visibility and engagement in detail [15]. The study also focused exclusively on YouTube, without examining cross‐platform diffusion across other social media ecosystems. Limited access to longitudinal subscriber and impression‐level data further restricted the ability to disentangle pre‐existing popularity from algorithmic amplification, despite evidence that recommendation systems can elevate content from smaller channels.

Future research should examine additional network properties, including density and betweenness centrality, to assess risks of message homogeneity and identify ‘bridge’ content connecting distinct audience communities. Longitudinal analyses of diffusion dynamics, including preferential attachment processes [56], and mixed‐methods approaches combining computational analysis with surveys or experiments would further advance understanding of how credibility, influence and behaviour interact in digital health communication.

## 5 Conclusion

This study demonstrated that the circulation of sleep‐health information on YouTube is shaped less by creator credibility and more by the sociotechnical dynamics of platformed communication. By integrating diffusion theory, social learning processes and network analysis, the study showed how influence emerges from the interaction of relational positioning, audience reinforcement and algorithmic amplification. These dynamics revealed a persistent credibility–influence gap, challenging assumptions within DOI and SCT that credibility is a reliable predictor of adoption in digital environments.

The findings highlighted the need to update theoretical models of diffusion and to design health communication strategies that engage with platform logics rather than work against them. Collaborations between public health organisations and high‐reach creators, enhanced digital literacy and more transparent credibility signals within recommendation systems may help mitigate the structural disadvantages faced by expert‐led sources. By foregrounding the role of platform infrastructures in shaping information exposure, this study contributed to ongoing discussions of platform governance, equity and public health in contemporary networked media environments.

## Author Contributions

**Atousa Ghahramani** conceived and led the study, developed the research questions and coordinated the overall project. Atousa Ghahramani and **Maria Prokofieva** designed the methodology and analytical approach. Atousa Ghahramani performed the data collection and analysis. Atousa Ghahramani drafted the original manuscript. Maria Prokofieva and **Maximilian Pangratius de Courten** contributed to the interpretation of the results and provided critical revisions to the manuscript for important intellectual content. All authors reviewed, approved the final version of the manuscript and agreed to be accountable for all aspects of the work.

## Funding

The authors have nothing to report.

## Ethics Statement

The study adhered to established ethical standards by analysing publicly available YouTube data, including metadata and user interactions, without collecting private or personally identifiable information. A waiver of informed consent was approved by the Victoria University Human Research Ethics Committee (HRE24‐088). All data were aggregated and anonymised to protect user privacy, in accordance with ethical guidelines for the use of online data and principles of transparent and responsible public health research.

## Conflicts of Interest

The authors declare no conflicts of interest.

## Data Availability

The data that supported the findings of this study were derived from publicly available sources on YouTube and collected using the YouTube Data API in accordance with the platform's terms of service. Due to platform restrictions and ethical considerations, raw data containing user‐generated content cannot be shared publicly. Aggregated data, network measures and analysis code supporting the results of this study are available from the corresponding author upon reasonable request.
